# Hearing Loss in Mucopolysaccharidoses: Current Knowledge and Future Directions

**DOI:** 10.3390/diagnostics10080554

**Published:** 2020-08-04

**Authors:** Jeremy Wolfberg, Keerthana Chintalapati, Shunji Tomatsu, Kyoko Nagao

**Affiliations:** 1 Nemours Biomedical Research, Nemours/Alfred I. duPont Hospital for Children, Wilmington, DE 19803, USA; jeremywolfberg@gmail.com (J.W.); keerthana@wustl.edu (K.C.); shunji.tomatsu@nemours.org (S.T.); 2 Department of Communication Sciences and Disorders, West Chester University, West Chester, PA 19383, USA; 3 Department of Biology, Washington University in Saint Louis, Saint Louis, MO 63130, USA; 4 Department of Pediatrics, Thomas Jefferson University, Philadelphia, PA 19107, USA; 5 Department of Pediatrics, Graduate School of Medicine, Gifu University, Gifu 501-1194, Japan; 6 Department of Pediatrics, Shimane University, Shimane 690-8504, Japan; 7 College of Health Sciences, University of Delaware, Newark, DE 19716, USA

**Keywords:** hearing loss, inner ear, middle ear, otitis media

## Abstract

Mucopolysaccharidoses (MPS) are a group of lysosomal storage disorders caused by a deficiency of one of the enzymes involved in the degradation of glycosaminoglycans. Hearing loss is a common clinical presentation in MPS. This paper reviews the literature on hearing loss for each of the seven recognized subtypes of MPS. Hearing loss was found to be common in MPS I, II, III, IVA, VI, and VII, and absent from MPS IVB and MPS IX. MPS VI presents primarily with conductive hearing loss, while the other subtypes (MPS I, MPS II, MPS III, MPS IVA, and MPS VII) can present with any type of hearing loss (conductive, sensorineural, or mixed hearing loss). The sensorineural component develops as the disease progresses, but there is no consensus on the etiology of the sensorineural component. Enzyme replacement therapy (ERT) is the most common therapy utilized for MPS, but the effects of ERT on hearing function have been inconclusive. This review highlights a need for more comprehensive and multidisciplinary research on hearing function that includes behavioral testing, objective testing, and temporal bone imaging. This information would allow for better understanding of the progression and etiology of hearing loss. Owing to the prevalence of hearing loss in MPS, early diagnosis of hearing loss and annual comprehensive audiological evaluations are recommended.

## 1. Introduction

Mucopolysaccharidoses (MPS) represent a group of rare lysosomal storage disorders. There are currently seven recognized subtypes of MPS caused by a deficiency of one of eleven enzymes involved in the degradation of glycosaminoglycans (GAGs), as shown in Table 1.

Most of the subtypes are caused by autosomal recessive inheritance, with only MPS type II being caused by X-linked recessive inheritance [8]. Both conductive and sensorineural hearing loss are common across the MPS subtypes. However, the literature focusing on hearing health in MPS is scarce, and many of the past studies are based on small samples. Large sample studies investigating enzyme replacement therapy (ERT) in MPS often report ERT effects on hearing, but detailed information is not available in general. Recently, there has been an increasing number of research papers focused on detailed hearing function. Below, we will review the literature on hearing loss in each MPS subtype. Table 2 shows a summary of references focused on audiological findings in patients with MPS.

## 2. Mucopolysaccharidosis Type I (Hurler Syndrome)

MPS type I (MPS I) is caused by a deficiency in the lysosomal enzyme alpha-L-iduronidase, which is involved in the degradation of dermatan sulfate (DS) and heparan sulfate (HS) [1]. MPS I was previously divided into three categories based on phenotype severity; that is, Hurler syndrome (OMIM 607014; the severe phenotype), Hurler–Scheie syndrome (OMIM 607015; the intermediate phenotype), and Scheie syndrome (OMIM 607016; the mild phenotype) [47]. Owing to overlapping symptoms in the three categories, MPS I is now categorized as severe, which includes Hurler syndrome, or attenuated, which includes Hurler–Scheie syndrome and Scheie syndrome [47,48,49]. The severe phenotype is most common, with a reported rate of 60.9% [47]. Neurological impairment is present in the severe phenotype, but is often absent from the attenuated phenotype [48]. Symptoms common in both phenotypes include coarse facial features, corneal clouding, hepatomegaly, cardiac valve abnormalities, hernia, lumbar kyphosis (gibbus), hearing loss, upper airway infection, and sleep apnea [47,48,49].

### 2.1. Types and Cause of Hearing Loss

Hearing loss is common in both the severe and attenuated phenotypes [49]. Reported rates of hearing loss range from 76.2% to 100% [10,20,21,22]. Hearing loss can present as conductive, sensorineural, or mixed [20]. Severity ranges from mild to severe, with mild and moderately severe being most common [10]. Chronic otitis media is also common, with an estimated rate of 89.1% [22]. The conductive component is attributed to frequent otitis media, a thickened tympanic membrane, and ossicular chain abnormalities [21,23,24,48]. The sensorineural component is believed to develop later in life after conductive hearing loss is already present [50]. Later development of sensorineural hearing loss was described in a MPS I murine model [26]. Inner ear abnormalities have been described in histopathological examination of patients with MPS I, but there is currently no consensus regarding the etiology of sensorineural hearing loss. Inner ear abnormalities that have been described include degeneration of the organ of Corti [23,24,27], damage to the stria vascularis [27], a significant decrease of both outer and inner cochlear hair cells [24], and damage to Reissner’s membrane [23,27]. A deposit of GAGs was also found on the vestibulo-cochlear nerve [23]. While cochlear and retrocochlear damage have been described, further research is needed to determine the precise etiology of the sensorineural component.

### 2.2. Efficacy of Clinical Treatments on Hearing

Hematopoietic stem cell transplantation (HSCT) and ERT are common treatments for MPS I. HSCT is primarily utilized to treat the severe phenotype, while ERT with laronidase is commonly utilized with the attenuated phenotype [47]. Several studies have described hearing improvement after HSCT [18,25]. It is recommended that HSCT occurs early in life, as improvement of sensorineural hearing loss was most significant in patients who received HSCT at 25 months or younger [18]. HSCT can be a dangerous treatment and has an estimated mortality rate of 11% [25]. ERT has been described as an effective method for changing the natural history of the MPS I attenuated form [51,52]. Studies have described that ERT does not improve audiological findings in patients with MPS I [21,53]. In a report of two case studies, a patient with conductive hearing loss experienced improved audiological findings after ERT, while a patient with mixed hearing loss did not exhibit audiological improvement [54]. Further research is needed to determine the effects of ERT on the conductive and sensorineural components of hearing loss. Particularly, the effects of ERT on inner ear function and the auditory neurophysiological responses at both brainstem and cortical levels would reveal detailed effects on sensorineural hearing function. Tympanostomy tubes are also commonly placed in patients with MPS I, with a reported rate of 78.2% [22]. Multiple tympanostomy tube placements are often required [19].

## 3. Mucopolysaccharidosis Type II (Hunter Syndrome)

MPS type II (MPS II), also known as Hunter syndrome (OMIM 309900), is the only subtype of MPS that is caused by X-linked recessive inheritance. Males are predominantly affected, but rare female cases have been reported [28,55]. Hunter syndrome is caused by a deficiency in the lysosomal enzyme iduronate-2-sulfatase [2], which is involved in the degradation of DS and HS [1]. MPS II is often divided into two categories (i.e., an attenuated or mild phenotype and a severe phenotype), but there is a wide spectrum of symptoms and phenotype severities [56]. The severe phenotype is characterized by early neurological impairment and intellectual disability, while the attenuated or mild phenotype only presents with mild neurological impairment [1,30]. Heart disease, skeletal deformities, upper respiratory tract infections, abdominal distention, developmental delays, and hearing loss are common in both phenotypes of MPS II [1,28,29,30,56].

### 3.1. Audiological Findings

Audiological findings have been well documented in MPS II. An MPS II international registry reported hearing loss data from 554 patients [28]. In a recent study of audiological findings of various types of MPS, 73.4% (91/124) of patients were diagnosed with MPS II [12]. Hearing loss is common, with reported rates ranging from 67.3% to 94%. Otitis media is also common, with a reported rate of 72.4% [28]. Hearing loss in MPS II has been described as progressive [12,15], with an estimated rate of loss at 1 dBHL per year [28]. Sensorineural hearing loss and mixed hearing loss are the most common types of hearing loss. The severity of hearing loss varies, with patients ranging from mild to profound [12,14,28]. Lack of cooperation has been cited as a barrier to determining the type and severity of hearing loss [12]. Owing to cognitive impairments, different modes of assessments, such as visual reinforcement audiometry, are often utilized to collect more accurate data [10]. Neurophysiological procedures such as otoacoustic emissions (OAEs) and auditory brainstem responses (ABRs) can be used in some cases to assess hearing function and provide timely clinical interventions to manage progressive hearing loss [57]. It is recommended that neurophysiological procedures be done without anesthesia, as MPS patients have a higher risk of complications from sedation [58,59,60].

### 3.2. Causes of Hearing Loss

Hunter syndrome presents with components of conductive and sensorineural hearing loss. Patients often first experience conductive hearing loss when they are younger. During childhood, sensorineural components emerge and lead to severe sensorineural hearing loss or mixed hearing loss. Owing to deposits of GAGs in the Eustachian tube and middle ear, frequent otitis media is common in patients with MPS II [9]. Accumulation of GAGs also leads to dysostosis of the ossicular chain and tympanic membrane scarring [61]. There is a lack of research on the sensorineural components in MPS II. Further research would be needed to determine if the sensorineural component is congenial or acquired owing to deposits of GAGs in the inner ear [9].

### 3.3. Efficacy of Clinical Treatments on Hearing

ERT has been described as a safe and effective treatment option for patients with MPS II [30]. ERT is most effective when started early in MPS II patients [61]. A murine study found that ERT was able to improve hearing levels and reduce the collection of GAGs in the outer and middle ear, but showed no effect on the inner ear [31]. Human research is needed on the efficacy of ERT for improving auditory function. Inserting tympanostomy tubes is an effective method for improving the conductive component of hearing loss by aerating the middle ear. Insertion of tympanostomy tubes has a reported rate of 49.6% [28]. Hearing aids are often utilized for patients with sensorineural hearing loss. Hearing aid use has a reported rate of 40.6% in patients with MPS II [28]. Recently, an adolescent patient with MPS II experienced hearing improvement after the implantation of a cochlear implant [62].

## 4. Mucopolysaccharidosis Type III (Sanfilippo Syndrome)

MPS type III (MPS III), also known as Sanfilippo syndrome, currently has four recognized subtypes (i.e., type A, B, C, and D), which are each caused by a deficiency to one of four enzymes that are responsible for the degradation of heparan sulfate, as shown in Table 3. Declined neurocognitive functions such as developmental delays, language delay, aggressive behaviors, and hearing loss are common among patients with all subtypes of MPS III [3,32,63,64,65], and their causes are often attributed to the degeneration of the central nervous system (CNS) [3]. Reported incidences and estimated prevalence vary by countries or regions of previous studies [66]. MPS IIIA and IIIB are common subtypes among the four, while MPS IIIC and IIID are rarer forms [66].

### 4.1. Audiological Findings

There is limited research focusing on the assessment of auditory function in human patients with MPS III. Only a handful of studies have reported clinical symptoms of patients with MPS IIIC and MPS IIID [3,67,68], and detailed descriptions of the progression of hearing loss are not well documented in these subtypes. Because MPS III affects the CNS, standard hearing assessment that requires patients’ compliance is challenging in many cases. Poor documentation of auditory function in MPS III may be owing to an inability to accurately collect data, as collecting audiometric data has been described as being difficult in patients with MPS III because of behavioral problems [3,67,68]. Previously reported rates of hearing loss are 87% (48/55) in MPS IIIA [32], 100% (3/3) in MPS IIIB [69], 75% (15/20) in MPS IIIC [67], and 25% (1/4) in MPS IIID [64]. The severity of hearing loss ranged from mild to moderate-severe in MPS IIIA [32] and moderate to severe in MPS IIIB [69]. Sensorineural hearing loss was the most common type of hearing loss [10,12,32,69], but the type of hearing loss was not determined in some patients because comprehensive hearing assessment was difficult to conduct or not available [65]. Conductive issues also appear early in life, as otitis media has a reported rate of 91%, and tympanostomy tubes are placed in most patients before 5 years [32]. While both conductive and sensorineural components have been shown to be present in patients with Sanfilippo syndrome, more research focused on audiometric findings and auditory function in human patients would be valuable in determining the need for hearing services to improve quality of life. Cortical potentials can be used to assess auditory processing abnormality in patients with impaired cognitive function.

### 4.2. Cause of Hearing Loss

A murine model of MPS IIIB displayed abnormalities in the middle ear, otitis media, hair cell loss in the inner ear, and damage to outer sulcus and pillar cells in the organ of Corti, affecting the base of the cochlea [31]. Chronic otitis media in MPS III contribute to the conductive hearing loss. It is likely that the disease simultaneously affects both the inner ear and the CNS, but further research is needed to understand the progression of hearing loss in MPS III.

### 4.3. Efficacy of Clinical Treatments on Hearing

Currently, there are no treatments available for MPS III. Severe impairment of the CNS has been described as a barrier for treatment development [70]. Bone marrow transplantation (BMT) and umbilical cord stem cell transplantation do not ameliorate the impairments to the CNS in MPS III [71,72,73]. ERT is also ineffective, as enzymes are not able to cross the blood–brain barrier [70]. The use of Genistein, an isoflavone, in substrate reduction therapy has been described as a safe and potentially effective method to reduce the collection of GAGs, but further research is needed to determine the therapeutic efficacy of genistein at improving neurological and auditory function [74]. Murine models have found that intracranial adeno-associated virus (AAV) gene therapy improved auditory function, as assessed by ABR, and reduced GAGs levels in the CNS, middle ear, and inner ear [75,76]. Similar results were also found in murine models that utilized a combination of intracranial AAV gene therapy and BMT [76]. Human research is needed to determine the efficacy of gene therapy and the combined use of gene therapy and BMT on neurological and auditory function.

## 5. Mucopolysaccharidosis Type IV (Morquio Syndrome)

MPS type IV (MPS IV), also known as Morquio syndrome, is divided into two subtypes; that is, MPS IVA (OMIM 253000) and MPS IVB (OMIM 253010). MPS IVA is caused by a deficiency in the lysosomal enzyme N-acetylgalactosamine-6-sulfate sulfatase, which is involved in the degradation of chondroitin-6-sulfate and keratan sulfate (KS) [4]. MPS IVB is caused by a deficiency in the lysosomal enzyme beta-galactosidase, which is involved in the degradation of KS [5]. Ninety-five percent of MPS IV patients present with MPS IVA [77]. MPS IVA presents with a wide range of phenotypes, ranging from an attenuated form to a severe form. Bone deformities to the knees, back-spine, chest, wrist, hips, legs, and ankles are common [78]. Other common symptoms include short stature, upper and lower airway obstruction, hearing loss, cardiac abnormalities, corneal clouding, and dental abnormalities [78,79,80,81]. MPS IVB presents with a milder phenotype than MPS IVA [82,83].

### 5.1. Hearing Loss

Hearing loss is common in MPS IVA [11,14,34,35], but patients with MPS IVB have been described as having normal hearing [34,82,84]. Limited research was found on the audiological assessment of patients with MPS IVA, but previous studies suggest that patients with MPS IVA experience hearing loss as the disease progresses. Reported rates of hearing loss range from 67% [11,14] to 94% [35]. Hearing loss can present as conductive, sensorineural, or mixed with severity ranging from mild to profound. Recurrent otitis media are also common in patients with MPS IVA. Similar to other types of MPS, conductive hearing loss often presents in younger patients, while sensorineural or mixed hearing loss develops later. The study by Riedner and Levin found that conductive hearing loss was present in all patients younger than 8 years old, while sensorineural or mixed hearing loss was found in older patients [35]. The conductive component is likely caused by recurrent otitis media and a collection of GAGs on the tympanic membrane and ossicular chain [34]. The etiology of the sensorineural component remains unknown. Thanks to absent distortion products otoacoustic emissions (DPOAEs) and decreased ABR, Nagao et al. described hair cell loss as a likely contributing factor to sensorineural hearing loss [34]. Animal models have also described the role of KS in the inner ear [36], but further research is needed to determine the etiology in humans. Recently, a relationship has been described between height (skeletal severity) and hearing loss severity in patients with MPS IVA [34]. It is recommended that patients receive an annual audiological assessment that includes both behavior and electrophysiological testing [34,80].

### 5.2. Efficacy of Clinical Treatments on Hearing

ERT with elosulfase alfa, or Vimizim, is a safe and commonly utilized therapy for patients with MPS IVA [85,86,87]. While hearing improvement after ERT was described in a case report [88], further research with a larger sample size is needed to determine the efficacy of ERT on improving hearing. HSCT has been described in MPS IVA case studies, but the impact on hearing was not assessed in any of these studies [87,89,90]. AAV gene therapy [91,92] and substrate reduction therapy [93] have been studied in animal models, but the impact of hearing has not been assessed. Tympanostomy tubes are placed in some patients to help alleviate the conductive component, with a reported rate of 33% in MPS IVA [78]. Recently, an MPS IVA post-lingual patient with severe to profound sensorineural hearing loss presented with hearing improvement after the implantation of a cochlear implant [94].

## 6. Mucopolysaccharidosis Type VI (Maroteaux–Lamy Syndrome)

MPS type VI (MPS VI), also known as Maroteaux–Lamy syndrome (OMIM 253200), is a rare autosomal recessive lysosomal storage disorder caused by the deficiency of arylsulfatase B (ARSB) [6]. The reduced or absent activity of this enzyme leads to the accumulation of GAGs in the lysosomes and a consequential decline in the function of multiple organ systems. Symptoms usually appear in early childhood and include macrocephaly, heart problems, upper respiratory infections, umbilical hernias, hepatomegaly, corneal clouding, chronic otitis media, and hearing loss [95].

### 6.1. Hearing Loss in MPS VI

There are a few studies that have specifically examined hearing issues in MPS VI [10,11,14]. Most of the previous studies reported hearing or ear problems in relation to the effects of ERT. Recurrent acute otitis media are a common problem in patients with MPS VI [96]. Most patients with MPS VI suffer from mild to moderate conductive hearing loss [10,11,37,96]. A recent retrospective study of five patients with MPS VI reported that, while conductive hearing loss is common, it seems to be temporary and can be treated through surgical interventions such as tympanostomy tube placement [97]. Recurrent otitis media with effusion persisted into adolescents, and multiple sets of tympanostomy tubes were common among the patients with MPS VI. It has been reported that some patients exhibit sensorineural or mixed hearing loss [10,14], but it is rare that patients with MPS VI have sensorineural hearing loss [98]. Although studies have reported audiological findings in MPS VI, the progression of hearing loss as well as their onset are not well-documented.

### 6.2. Efficacy of Clinical Treatment on Hearing

The standard treatment for hearing issues in patients with MPS VI is tympanostomy tube placement to reduce recurrent otitis media [96]. However, tympanostomy tube placement may serve only as a temporary solution to middle ear issues, as the tubes lose function and fall out after a few years. Without the presence of any otolaryngologic interventions such as tympanostomy tube placement, hearing issues can progress and lead to severe or permanent hearing loss [97]. As for the ERT effects on hearing in patients with MPS VI, the results are still inconclusive [37,38,39,40,53]. Previous studies have indicated an unchanging hearing status after ERT in some patients, whereas other studies have indicated hearing improvement [38,39,40], even in the patient with sensorineural hearing loss. It is not clear how ERT improved sensorineural hearing loss in MPS VI. ERT could reduce upper respiratory infections and ear infections, and subsequentially reduce the occurrence of ear infections and prevent conductive hearing loss.

## 7. Mucopolysaccharidosis Type VII (Sly Syndrome)

MPS type VII (MPS VII), also known as Sly syndrome (OMIM 253220), is one of the rarer forms of MPS, with an estimated incidence of 1:300,000 to 1:2,000,000 live births [8,41]. Sly syndrome is caused by a deficiency in the lysosomal enzyme glucuronidase, which is involved in the degradation of chondroitin sulfate, DS, and HS [1]. Skeletal dysplasia, cognitive impairments, heart abnormalities, and hearing loss are common in patients with MPS VII [1,41,45,99].

### 7.1. Causes of Hearing Loss

Murine models of MPS VII have displayed abnormalities in the middle and inner ear. The conductive component has been attributed to cerumen impaction, recurrent otitis media, and ossicle articular alterations [45]. Inner ear pathology in a murine model suggested that the sensorineural component is attributed to the assembly of GAGs, severely affecting inner ear structures such as Reissner’s membrane, spiral limbus, spiral ligament, spiral prominence, and spiral ganglion [42]. Sensorineural deficits were observed at a later onset than conductive deficits.

### 7.2. Audiological Findings

Limited research was found focusing on the assessment of auditory function in human patients with MPS VII. This may be because of the low incidence and rare nature of Sly syndrome. Recent studies on the audiological assessment of various types of MPS have not included any participants with MPS VII [10,11,12,14]. In a physician’s medical history survey of 56 patients with Sly syndrome, sensorineural hearing loss was present in 41% of participants [41]. A case study presents audiometric results from a patient with a rare and milder phenotype, as the patient lived to the age of 52 years [100]. At the age of 13, this patient was described as having mixed bilateral hearing loss, with hearing thresholds determined to be 50 dB Hearing Level (dB HL) in the right ear and 40 dB HL in the left ear [100]. As this is a rare and milder phenotype, this may not be representative of typical thresholds and audiological diagnoses. Several murine models investigating the effects of various therapies have described audiological findings in mice with MPS VII. In a murine study investigating the effects of syngeneic BMT, the untreated murine model presented with an ABR that was 42 dB higher than the normal untreated mice [44]. A murine study investigating the effects of ERT states that a stimulus greater than 60 dB was needed at every frequency tested in order to elicit an ABR waveform response [43].

### 7.3. Efficacy of Clinical Treatment on Hearing

Murine models have been utilized to observe the efficacity of several therapy methods on the improvement of audiometric results and pathological findings [43,44,101]. Syngeneic BMT improved ABR to within normal limits at 11 weeks of age, reduced the severity of hearing loss at 33 weeks, and decreased the severity of otitis media and pathological abnormalities in a murine model [44,101,102]. ERT initiated at birth in mice led to the improvement in ABR thresholds, reduction in ossicular abnormalities, and a decrease in middle ear inflammation [43]. Treatment is described as being more effective when initiated at birth in both BMT and ERT [101]. Recently, the ERT vestronidase alfa, or Mepsevii, was approved for MPS VII in humans [103,104], but current research has not included audiological findings. Human research is needed to determine the efficacy of BMT and ERT in reducing hearing loss and audiological pathology.

## 8. Mucopolysaccharidosis Type IX (Natowicz Syndrome)

MPS type IX (MPS IX), also known as Natowicz syndrome, is an extremely rare autosomal recessive disorder that causes hyaluronidase deficiency (OMIM 601492). Currently, only four patients have been reported to have MPS IX and the corresponding hyaluronan accumulation [7]. The first report of MPS IX was in 1996, where a 14-year-old patient exhibited short stature and multiple tissue masses. Upon examination of this patient, a deficiency of hyaluronidase was identified [7,104,105]. The other three reported cases of MPS IX were within a single consanguineous family, where all patients exhibited knee or hip pain and joint swelling [46].

### 8.1. Hearing Issues

Unlike other MPS types, patients with MPS IX do not exhibit hearing loss among the few known reported cases [7,46]. Although it was reported that the first patient with MPS IX had frequent episodes of otitis media, the patient did not exhibit hearing loss or any speech and language issues [7]. No hearing issues were found in the other three cases of MPS IX [46].

### 8.2. Animal Models

Although there is only a small number of reported cases, animal models can give us insight on varied clinical symptoms and treatment options for patients with MPS IX. MPS IX is caused by the deficiency of hyaluronidase 1 (HYAL1) [105]. Murine models suggest that HYAL1 deficiency leads to joint pathology [106]. HYAL1-null mice had normal appearance, fertility, and tissue morphology. Osteoarthritis was found to be a primary indicator of HYAL1 deficiency [106,107]. Murine models have also shown increased expression of the hyaluronidase 3 (HYAL3) gene in the liver and the testes of HYAL1-null mice [106,107]. It is proposed that the HYAL3 gene may compensate HYAL1 deficiency in MPS IX [106]. Furthermore, animal models suggest that there may be a new subtype of MPS in humans that has not been identified [106,107,108]. Mice with hyaluronidase 2 (HYAL2) deficiency developed skeletal defects and cardiac anomalies [108].

## 9. Discussion

This article provides a review of the literature on audiological findings in patients with each of the recognized subtypes of MPS. According to the current literature, hearing loss is common in most of the subtypes of MPS; that is, MPS I (Hurler syndrome), MPS II (Hunter syndrome), MPS III (Sanfilippo syndrome), MPS IVA (Morquio syndrome type A), MPS VI (Maroteaux–Lamy syndrome), and MPS VII (Sly syndrome). Hearing loss is not present in patients with MPS IVB (Morquio syndrome type B) or MPS IX (Natowicz syndrome), although this is based on a small number of patients owing to the rarity of these subtypes. MPS VI is the only subtype that presents primarily with conductive hearing loss. The other subtypes (MPS I, MPS II, MPS III, MPS IVA, and MPS VII) can present with any type of hearing loss (conductive, sensorineural, or mixed hearing loss). In these subtypes, patients often first present with conductive hearing loss and later develop a sensorineural component, leading to sensorineural or mixed hearing loss. It is unclear why MPS VI is the only subtype that does not affect a sensorineural component in general. Sensorineural hearing loss has been reported in some rare cases with MPS VI, although it is not clear whether sensorineural hearing loss is owing to relatively slow progression of MPS VI. While CNS impairment is absent from patients with MPS VI, it is also absent from patients with MPS IVA [109], a subtype that presents with sensorineural and mixed hearing loss. Further research is needed to determine if there are pathophysiological differences between MPS VI and the other subtypes.

**Figure 1:**
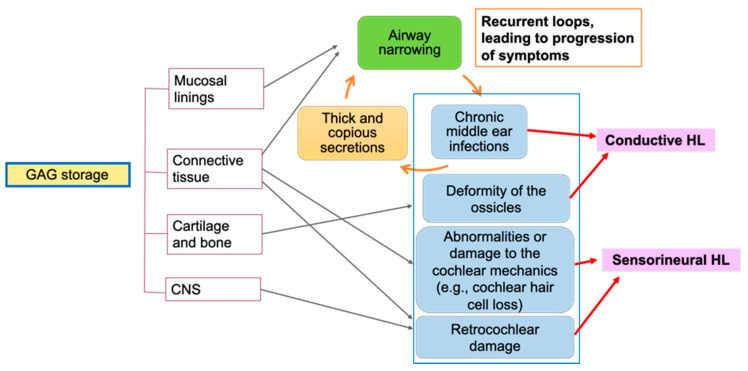
Hearing loss pathophysiology in mucopolysaccharidoses (MPS). GAG, glycosaminoglycan; CNS, central nervous system; HL, hearing loss.

More comprehensive audiological and pathophysiological research is needed to develop a more precise understanding of the progression of hearing loss in each MPS subtype. In particular, understanding both onsets and etiology of sensorineural hearing loss is critical to provide appropriate clinical intervention to patients. Figure 1 illustrates pathophysiology of hearing loss in MPS. This figure provides a broad overview of the pathophysiology, as the progression of hearing loss differs depending on the MPS subtype. Deposits of GAGs in mucosal linings, connective tissue, cartilages, bones, and the CNS lead to many of the components contributing to hearing loss. Chronic otitis media or persistent middle ear effusion is common in all subtypes beyond early childhood and contributes to the conductive component of hearing loss. Susceptibility to viral infections in patients with MPS is likely the main cause of frequent middle ear effusion, which could lead to structural changes in the tympanic membrane and ossicular deformities. A vicious cycle present between airway narrowing, chronic otitis media, and collections of thick secretions is believed to further the progression of the conductive component. Tympanostomy tubes are effective at treating middle ear dysfunction, but multiple tube replacements are common in MPS. This can lead to tympanic membrane thickening and scaring. Tympanic membrane thickening and ossicular chain abnormalities have been described as a contributing factor to conductive hearing loss in MPS I [21,23], MPS II [61], MPS IVA [34], and MPS VII in a murine model [45]. There is currently no consensus on the etiology of the sensorineural component for any of the recognized subtypes. A reduction in outer and inner cochlear hair cells likely contributes to the sensorineural component, as this has been described in MPS I [24], MPS IV [34], and MPS IIIB in a murine model [44]. The accumulation of GAGs is believed to cause damage to structures in the inner ear. Abnormalities to the organ of Corti, Reissner’s membrane, stria vascularis, and vestibulo-cochlear nerve have been described [20,21,23,24,30,41], but there is no consensus on their prevalence, severity, or effect on sensorineural hearing loss. Hearing loss does not seem to appear only at certain frequency ranges among patients with MPS, yet more detailed analysis of hearing loss would be crucial to understand the progression of sensorineural hearing loss. While a collection of GAGs has been shown to cause cochlear and retrocochlear damage, more research is needed to determine a more precise onset and etiology of sensorineural hearing loss. It is suspected that disease severity and sensorineural hearing loss are correlated with each other, but the relationship between hearing test results and other clinical measures such as GAG levels or bone density measures has not been studied. Owing to the complex nature of hearing loss in MPS, multidisciplinary research studies including experts in audiology, otolaryngology, biochemistry, neurology, radiology, and genetics are recommended.

Treatment options vary depending on the MPS subtype. ERT is the most common treatment for MPS, with approved therapies available for [51,52], MPS II [30], MPS IVA [85,86,87], MPS VI [37], and MPS VII [103,104]. Research on the effects of ERT on audiological function has been inconclusive [53]. While case reports on MPS IVA [88] and MPS VI [38,39] have described hearing improvement after ERT, studies on MPS I [21] and MPS VI [37] have described no change in audiological function after ERT. A MPS II murine model found that ERT was able to improve the conductive component, but was unable to ameliorate the sensorineural component [31]. This was also exhibited in two patients with MPS I, where the patient with conductive hearing loss presented with improved hearing after ERT and the patient with mixed hearing loss did not experience any audiological improvement [54]. More comprehensive audiological research is needed to delineate the effects of ERT on the conductive and sensorineural components for each MPS subtype. HSCT has resulted in improved audiological function in MPS I [18,25], but the impact of hearing has not been studied in MPS IVA [87,89,90]. Owing to the high prevalence of hearing loss in most MPS subtypes, future studies on treatment efficacy should include comprehensive audiological evaluation.

This review highlights the need for more comprehensive audiological research for all subtypes of MPS. Previous studies on hearing loss have primarily utilized behavioral audiological testing and tympanometry. In addition to commonly reported pure tone average threshold levels, future research should report frequency specific hearing thresholds. Frequency specific information (e.g., averages of multiple audiograms from a given MPS subtype) would provide valuable insights into specific cochlear regions affected by MPS. OAE and ABR have occasionally been utilized, but these studies often do not provide detailed results. Temporal bone imaging is rarely utilized in the current literature. In addition to common behavioral audiological assessment, future research should utilize auditory neurophysiological assessment and temporal bone imaging as a part of a comprehensive assessment. A recent paper by van den Broek et al. is a prime example of a comprehensive assessment of auditory function [19]. This retrospective review on the effects of hematopoietic cell transplantation on hearing loss utilized pure tone audiometry, neurophysiological assessment (ABR), and imaging. Owing to respiratory, skeletal, pulmonary, and cardiovascular abnormalities, MPS patients have an increased risk of sedation complications [58,59,60]. Because patients with MPS undergo radiological and neuroradiological imaging procedure for their skeletal issues, it is desirable to obtain temporal bone imaging to assess the ear structures without increasing risk of anesthesia. OAE and ABR can be conducted without general anesthesia. Comprehensive audiological research would provide a better understanding of the progression and etiology of hearing loss in each subtype of MPS. Identifying the etiology of the sensorineural hearing loss common in many patients with MPS would help clinicians to determine optimal clinical interventions precisely prescribed to each patient. A better understanding of the progression of hearing loss could also be used in the assessment of clinical intervention efficacy.

Finally, this review highlights the importance of early diagnosis of hearing loss and annual comprehensive audiological evaluation for patients with MPS. This is consistent with other recommendations found in the literature [10,11,12,13,14,15,16,17,19,34,80,110,111,112]. Owing to the high prevalence and progressive nature of hearing loss in MPS, regular audiological assessments are needed to determine the progression of hearing loss and the need for hearing aids or tympanostomy tube placement. Early detection and intervention can help in the improvement of quality of life for patients with MPS.

## Figures and Tables

**Table 1:** Mucopolysaccharidoses (MPS) subtypes with enzyme deficiency and subsequent collection of glycosaminoglycans (GAGs).

MPS Subtype	Enzyme	GAGs
MPS I (Hurler syndrome)	alpha-L-iduronidase [1]	DS and HS [1]
MPS II (Hunter syndrome)	iduronate-2-sulfatase [2]	DS and HS [1]
MPS IIIA (Sanfilippo syndrome type A)	heparan N-sulfatase [3]	HS [1]
MPS IIIB (Sanfilippo syndrome type B)	alpha-N-acetylglucosaminidase [3]	HS [1]
MPS IIIC (Sanfilippo syndrome type C)	acetyl CoA alpha-glucosaminide acetyltransferase [3]	HS [1]
MPS IIID (Sanfilippo syndrome type D)	N-acetylglucosamine 6-sulfatase [3]	HS [1]
MPS IVA (Morquio syndrome type A)	N-acetylgalactosamine-6-sulfate sulfatase [4]	Chondroitin-6-sulfate and KS [4]
MPS IV B (Morquio syndrome type B)	beta-galactosidase [5]	KS [5]
MPS VI (Maroteaux–Lamy syndrome)	arylsulfatase B [6]	DS [1]
MPS VII (Sly syndrome)	glucuronidase [1]	Chondroitin sulfate, DS, and HS [1]
MPS IX (Natowicz syndrome)	hyaluronidase [7]	Hyaluronic acid [7]

DS = dermatan sulfate; HS = heparan sulfate; KS = keratan sulfate.

**Table 2:** Summary of studies on auditory characteristics of MPS.

Source	MPS Type(s)	Article Type (Research, Case Study, Review)	N	Animal/Human
Simmons et al., 2005 [9]	All MPS types	Retrospective review	N/A	Human
Silveira et al., 2018 [10]	I, II, III, IV, VI	Descriptive, cross-sectional study	53	Human
Lenka et al., 2020 [11]	I, II, III, IV, VI	Retrospective review	61	Human
Ahn et al., 2019 [12]	I, II, III, IV, VI	Retrospective review	124	Human
Mesolella et al., 2013 [13]	I, II, III, IV, VI	Observational Study	20	Human
Lin et al., 2014 [14]	I, II, IV, VI	Clinical study	39	Human
Vargas-Gamarra et al., 2017 [15]	I, II, III, IV	Retrospective study	23	Human
Gokdogan et al., 2016 [16]	I, III, IV, VI	Clinical study	9	Human
Giraldo et al., 2020 [17]	II, IVA, VI	Retrospective study	35	Human
Da Costa et al., 2012 [18]	I, II	Retrospective study	30	Human
van den Broek et al., 2020 [19]	I, VI	Retrospective study	32	Human
Aldenhoven et al., 2015 [20]	I	Retrospective study	217	Human
Dualibi et al., 2016 [21]	I	Prospective study	9	Human
Kiely et al., 2017 [22]	I	Retrospective review	55	Human
Friedmann et al., 1985 [23]	I	Histopathological study	2	Human
Kariya et al., 2012 [24]	I	Temporal bone scan study	6	Human
Souillet et al., 2003 [25]	I	Prospective study	27	Human
Schachern et al., 2007 [26]	I	Research study	N/A	Mouse
Schachern et al., 1984 [27]	I	Temporal bone scan study	3	Human
Keilmann et al., 2012 [28]	II	Survey/Registry	554	Human
Chiong et al., 2017 [29]	II	Case series	23	Human
Muenzer et al., 2006 [30]	II	Clinical trial	96	Human
Hong et al., 2012 [31]	II	Research study	N/A	Mouse
Buhrman et al. 2014 [32]	IIIA	Retrospective review	46	Human
Heldermon et al., 2007 [33]	IIIB	Research study	N/A	Mouse
Nagao et al., 2018 [34]	IVA, IVB	Clinical study	14	Human
Riedner and Levin, 1977 [35]	IV	Audiological/Otologic review	21	Human
Swartz and Santi, 1997 [36]	IV	Animal research	N/A	Animal (chinchilla, cat, gerbil, rabbit)
Gomes et al., 2019 [37]	VI	Clinical review	362	Human
Furujo et al., 2017 [38]	VI	Case study	2	Human
Harmatz et al., 2014 [39]	VI	Clinical trial review	N/A	Human
Horovitz et al., 2013 [40]	VI	Retrospective review	34	Human
Montaño et al., 2016 [41]	VII	Survey	56	Human
Ohlemiller et al., 2002 [42]	VII	Research study	N/A	Mouse
O’Connor et al., 1998 [43]	VII	Research study	N/A	Mouse
Sands et al., 1995 [44]	VII	Research study	N/A	Mouse
Berry et al., 1994 [45]	VII	Research study	N/A	Mouse
Natowicz et al., 1996 [7]	IX	Case report	1	Human
Imundo et al., 2011 [46]	IX	Clinical case reports	3	Human

**Table 3:** Genes and affected enzymes of MPS III.

Type	Enzyme	OMIM Number	Gene
MPS IIIA	Heparan N-sulfatase	252900	*SGSH*
MPS IIIB	Alpha-N-acetylglucosaminidase	252920	*NAGLU*
MPS IIIC	Acetyl CoA alpha-glucosaminide acetyltransferase	252930	*HGSNAT*
MPS IIID	N-acetylglucosamine 6-sulfatase	252940	*GNS*

## Abbreviations

MPS	Mucopolysaccharidosi(e)s
GAGs	Glycosaminoglycans
ERT	Enzyme replacement therapy
dB HL	dB hearing level
DS	Dermatan sulfate
HS	Heparan sulfate
HSCT	Hematopoietic stem cell transplantation
OAE	Otoacoustic emissions
ABR	Auditory brainstem response
CNS	Central nervous system
BMT	Bone marrow transplantation
AAV	Adeno-associated virus
KS	Keratan sulfate
DPOAE	Distortion products otoacoustic emissions
ARSB	Arylsulfatase B
HYAL1	Hyaluronidase 1
HYAL3	Hyaluronidase 3
HYAL2	Hyaluronidase 2
HL	Hearing loss
